# Fibular head osteotomy: A new technique for better exposure of postero-lateral tibial plateau fracture

**DOI:** 10.1186/s10195-025-00836-z

**Published:** 2025-03-19

**Authors:** Shaozheng Yang, Yong Lian, Li Yang, Sushuang Ma, Chao Ding, Feng Huang, Yongqiang Liu, Heng Li, Zhu Mutan, Hua Zhong, Hongfen Chen

**Affiliations:** 1 Department of Orthopaedics, The Fifth Affiliated Hospital, Southern Medical University, Guangzhou, Guangdong China; 2 Guangzhou University of Chinese Medicine, No. 261, Longxi Avenue, Liwan District, Guangzhou, Guangdong China; 3 Loudi Vocational and Technical College, Loudi, Hunan China

**Keywords:** Tibial plateau fracture, Posterolateral fracture, Fibular head osteotomy

## Abstract

**Objective:**

Various osteotomy techniques have been explored for exposing the posterolateral tibial plateau in previous studies. However, these methods are often complex, cause significant damage to normal anatomical structures, compromise knee joint stability, and pose risks to knee function, thus limiting their clinical application. This study proposes a new fibular head osteotomy technique for treating posterolateral tibial plateau fractures, aiming to achieve favorable surgical outcomes.

**Methods:**

Thirteen patients who underwent surgical treatment for posterolateral tibial plateau fractures between March 2020 and August 2023 at our hospital were included in this study. The study was approved by the clinical ethics committee of our institution. All patients provided informed consent before participation. Surgeries were performed through a modified Frosch approach combined with partial fibular head osteotomy, while preserving part of the biceps femoris tendon attachment to the fibula. Postoperative fracture reduction quality was assessed using X-rays and computed tomography (CT) scans, in accordance with the Rasmussen radiology scoring system. Knee joint function was evaluated at the final follow-up using the Hospital for Special Surgery (HSS) scoring system. The healing of the fibular head osteotomy site and the presence of any complications were also assessed.

**Results:**

All 13 patients were followed up with for an average of 12.2 months (range: 9–17 months). All fractures, collapse, and deformities were corrected. The mean Rasmussen radiological score was 15.5 ± 2.5 (range: 10–18), with four cases rated as excellent, eight as good, and one as fair. The mean Hospital for Special Surgery (HSS) score was 89.8 ± 6.4 (range: 78–98), with 10 cases rated as excellent and 3 as good. No posterolateral knee instability was observed during physical examination at the final follow-up. There were no complications such as surgical site infection or common peroneal nerve injury.

**Conclusions:**

Partial fibular head osteotomy combined with preservation of the biceps femoris tendon attachment is an effective technique for treating posterolateral tibial plateau fractures. This method allows for successful fracture reduction and fixation without compromising knee joint function.

## Introduction

Posterolateral (PL) tibial plateau fractures account for approximately 7–15% of all tibial plateau fractures [1, 2]. This specific type of fracture is typically caused by flexion or flexion–external rotation forces on the knee joint, primarily involving the posterior (P) one-third of the lateral tibial plateau [3]. Posterolateral tibial plateau fractures often result in significant articular surface collapse. If the fracture deformity is not corrected, it may lead to post-traumatic arthritis and knee instability [4]. Numerous studies have reported various surgical approaches for the reduction and fixation of posterolateral tibial plateau fractures, including both osteotomy and non-osteotomy approaches [1, 5–9]. When using a non-osteotomy approach, the fibular head serves as a bony obstruction to the posterolateral tibial plateau, with a maximum obstruction rate of up to 61.7 ± 4.9% [10]. To expose the articular surface, it is often necessary to enlarge the incision or apply excessive traction during surgery, increasing tissue trauma and the risk of common peroneal nerve injury [1, 5]. Osteotomy approaches include fibular neck osteotomy, lateral femoral condyle osteotomy, and Gerdy’s tubercle osteotomy [6–9]. While these approaches significantly improve exposure of the posterolateral tibial plateau articular surface, they also have notable drawbacks, such as greater tissue trauma, a higher risk of common peroneal nerve injury, disruption of normal knee anatomy, potential knee instability, and insufficient exposure in some cases [11, 12].

In response to these challenges, we propose a partial fibular head osteotomy technique that preserves the biceps femoris tendon attachment for the treatment of posterolateral tibial plateau fractures. In this paper, we will introduce the details of this technique and report its early clinical outcomes.

## Methods

This study included 13 patients who underwent surgical treatment for posterolateral tibial plateau fractures at our hospital between March 2020 and August 2023. The study was approved by the institutional ethics committee, and all patients provided written informed consent before participation. Inclusion criteria were diagnosis of an acute comminuted posterolateral tibial plateau fracture with joint surface collapse > 2 mm, age ≥ 18 years with radiographic evidence of closed epiphyseal plates, and capability to complete follow-up. Exclusion criteria were pathological fractures, multiple fractures, open fractures, fractures involving the fibular head, fractures with neurovascular injuries, fractures treated using alternative surgical approaches, and severe knee osteoarthritis. Preoperative assessments included standard anteroposterior and lateral X-rays of the knee, as well as computed tomography (CT) scans with three-dimensional (3D) reconstruction for fracture classification and severity evaluation. Fractures were classified using the Kfuri–Schatzker system, an evolution of the three-column classification by Luo Congfeng and the Schatzker classification [13, 14] (Table 1). Surgical exposure of the posterolateral tibial plateau was achieved using a modified Frosch approach [15] combined with a partial fibular head osteotomy while preserving the biceps femoris tendon attachment. After reduction of the fracture fragments, supportive fixation was achieved using a buttress plate. Postoperative anteroposterior and lateral X-rays, as well as CT scans with three-dimensional reconstruction, were performed on the second day after surgery to assess reduction quality. Fracture reduction was evaluated using the Rasmussen radiology scoring system [16], which assesses articular surface compression, tibial plateau widening, and varus or valgus deformities. Scores of 18 indicate excellent, 12–17 good, 6–11 fair, and < 6 poor outcomes. At the final follow-up, knee joint function was assessed using the Hospital for Special Surgery (HSS) scoring system [17]. This scoring system evaluates pain, walking and standing function, range of motion, and muscle.

**Table 1:** Patient demographics

Patients	Sex	Age (years)	Injury mechanism	Kfuri–Schatzker
1	Female	54	Traffic accident	Type V, PL + PM
2	Female	38	Traffic accident	Type III, PL
3	Female	50	Fall from height	Type V, P + PM
4	Male	42	Traffic accident	Type II, PL
5	Female	44	Traffic accident	Type V, PL + PM + AM
6	Male	64	Other injuries	Type III, PL
7	Male	30	Other injuries	Type III, P
8	Male	36	Other injuries	Type V, P + PM
9	Male	42	Traffic accident	Type V, PL + PM
10	Male	55	Traffic accident	Type III, PL
11	Female	57	Traffic accident	Type II, PL
12	Female	49	Other injuries	Type V, PL + PM + AM
13	Female	42	Traffic accident	Type III, PL

*P* posterior, *PL* posterolateral, *AM* anteromedial, *PM* posteromedial

### Surgical techniques

#### Incision and exposure

**Fig. 1:**
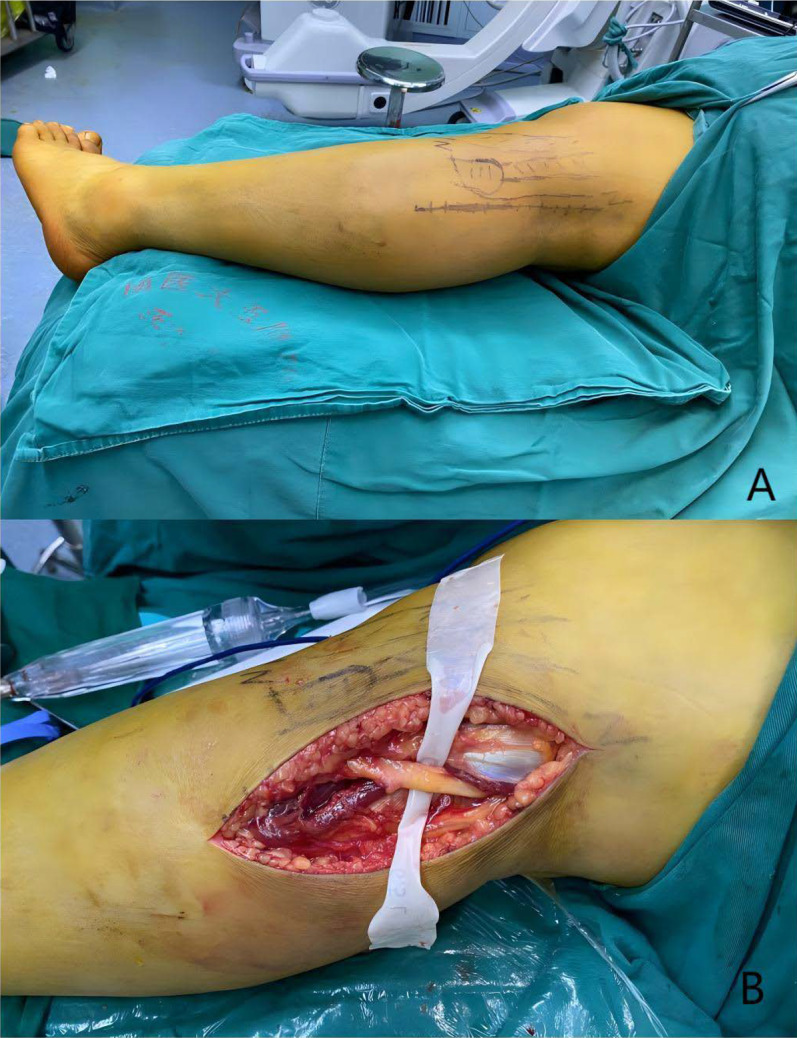
Patient positioning, incision selection, and dissection of the common peroneal nerve during surgery. **A** The patient is positioned in the lateral decubitus position with the operative limb placed superiorly. **B** The surgical incision is a straight line, originating approximately 3 cm proximal to the joint line and extending 5–7 cm distally along the posterior edge of the fibular head. The common peroneal nerve is exposed, carefully dissected, retracted, and protected during the procedure

All surgical procedures were performed by the same lead surgeon. After patients were administered spinal or general anesthesia, their position was determined on the basis of the presence or absence of medial column fractures, with either a lateral or prone position employed accordingly. A straight incision was made, originating approximately 3 cm proximal to the joint line and extending 5–7 cm distally along the posterior edge of the fibular head. The common peroneal nerve was exposed along the posterior edge of the biceps femoris tendon, carefully dissected and released, and gently protected throughout the procedure (Fig. 1). The lateral head of the gastrocnemius was separated from the soleus muscle and retracted medially to expose and ligate the inferior lateral genicular vessels. The soleus muscle was partially transected along the posterior edge of the fibula, and the posterolateral origin of the tibia was subperiosteally dissected. These structures were retracted medially along with the lateral head of the gastrocnemius. The popliteus tendon was exposed, marked if necessary, and transected during the procedure (to be repaired after fracture reduction and fixation). The posterior joint capsule was incised and subperiosteal dissection performed, with the distal extent limited to within 5 cm of the joint line to avoid damaging branches of the anterior tibial artery.

#### Osteotomy and fixation

**Fig. 2:**
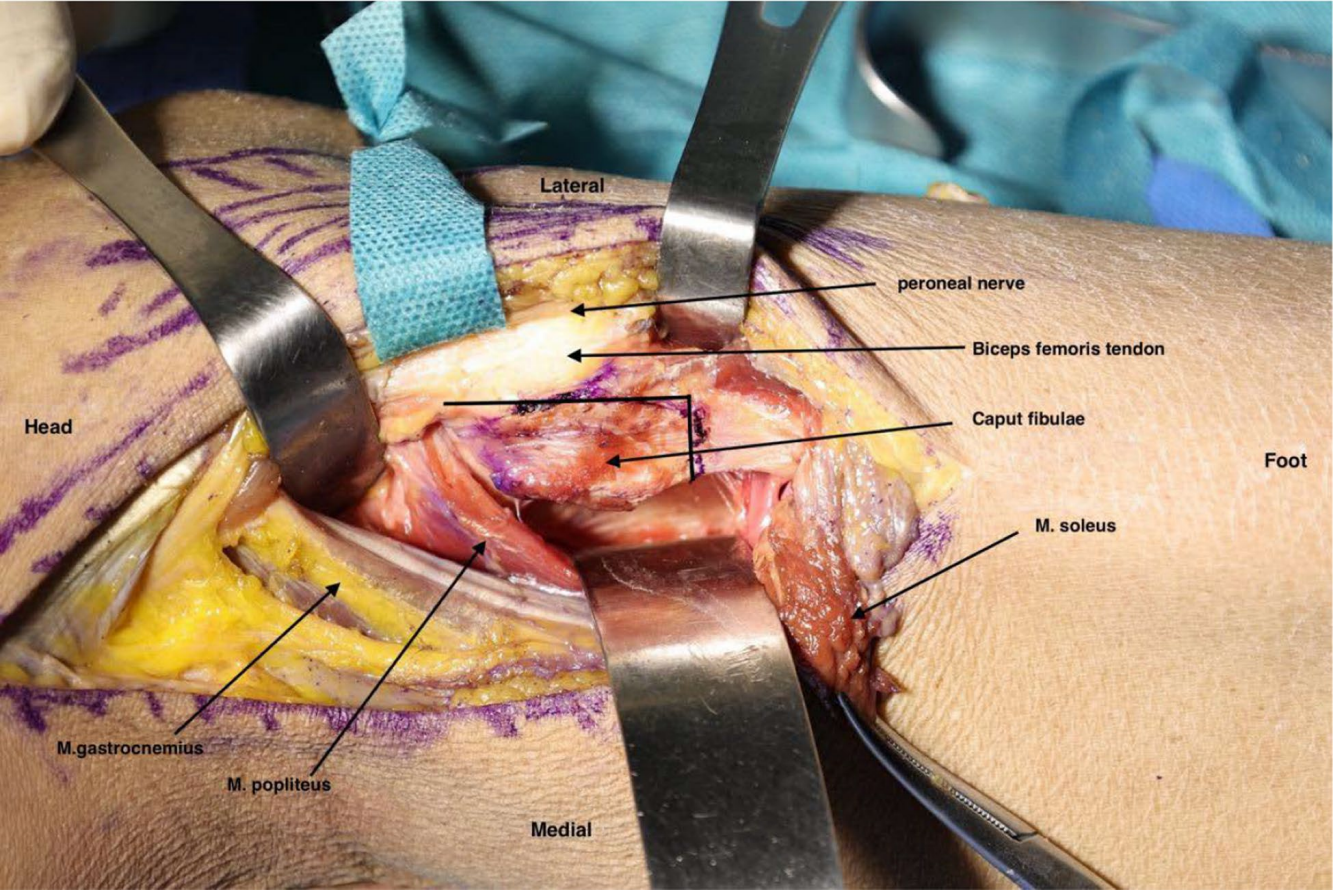
The diagram illustrates an osteotomy procedure. The peroneal nerve is retracted anteriorly, and the *musculus soleus*, or soleus muscle is detached from the fibular head. The *musculus gastrocnemius* and popliteus tendon are retracted posteriorly. A line is drawn along the posterior border of the biceps femoris tendon insertion to the fibular neck. A perpendicular line is then made at the fibular neck–shaft junction. The region defined by the intersection of these two lines constitutes the osteotomy zone

**Fig. 3:**
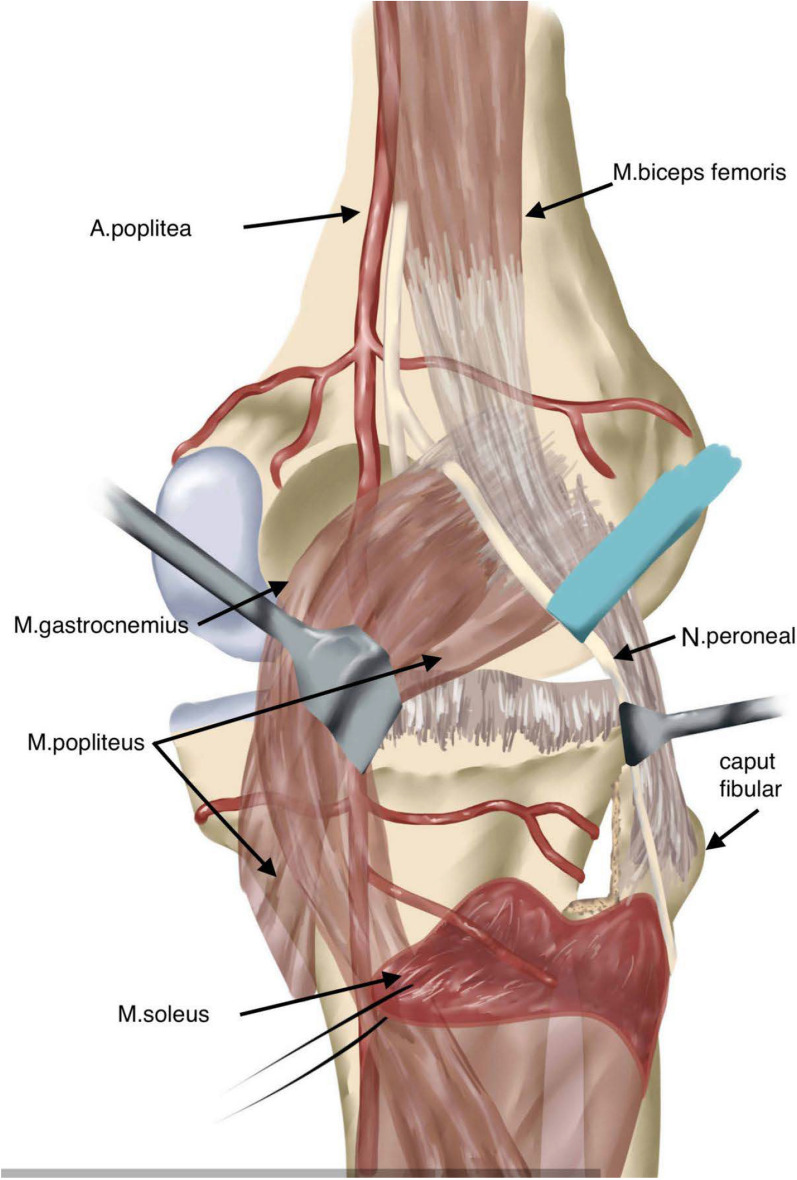
An illustration of the posterolateral corner of the tibia. The L-shaped fibular head osteotomy provides complete exposure of the posterolateral tibial plateau, enabling optimal visualization for fracture reduction and fixation

**Fig. 4:**
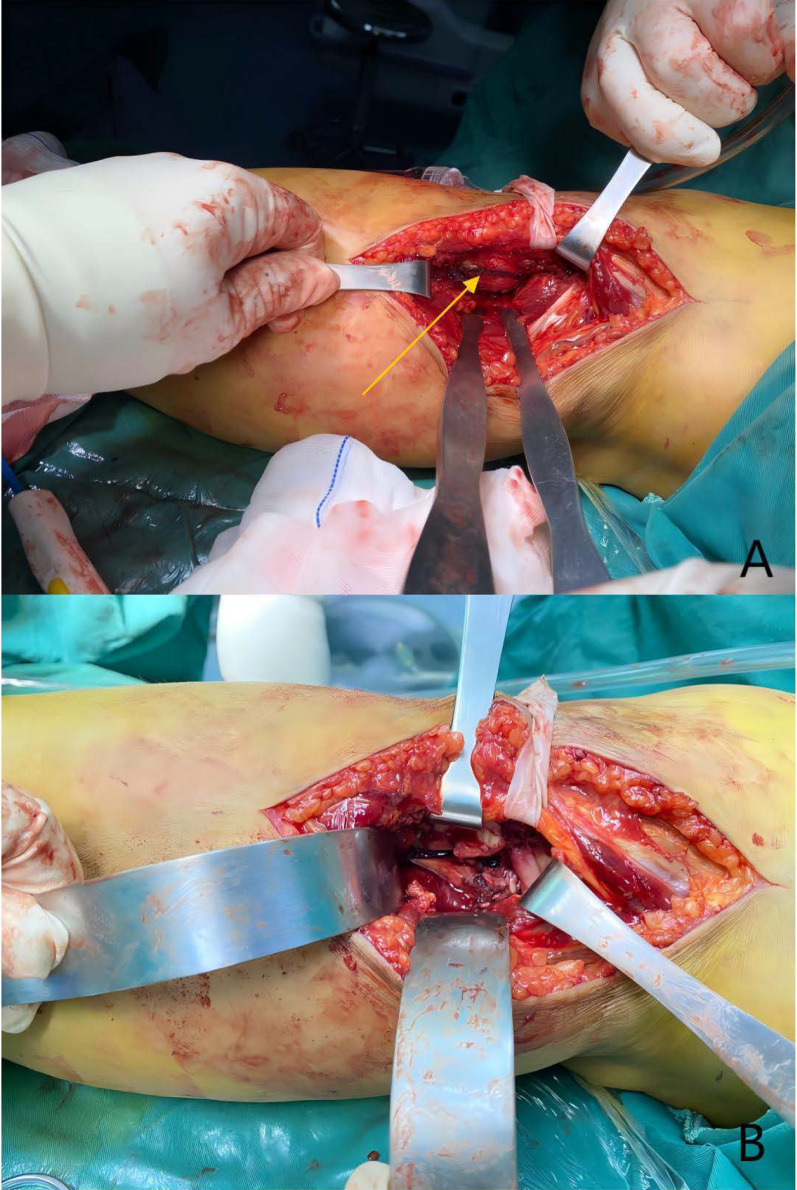
Posterolateral articular surface and fracture region of the tibial plateau. **A** Before the osteotomy, visualization of the posterolateral articular surface is obstructed by the fibular head (indicated by the yellow arrow). **B** After partial fibular head osteotomy, the posterolateral articular surface and fracture region are fully exposed

**Fig. 5:**
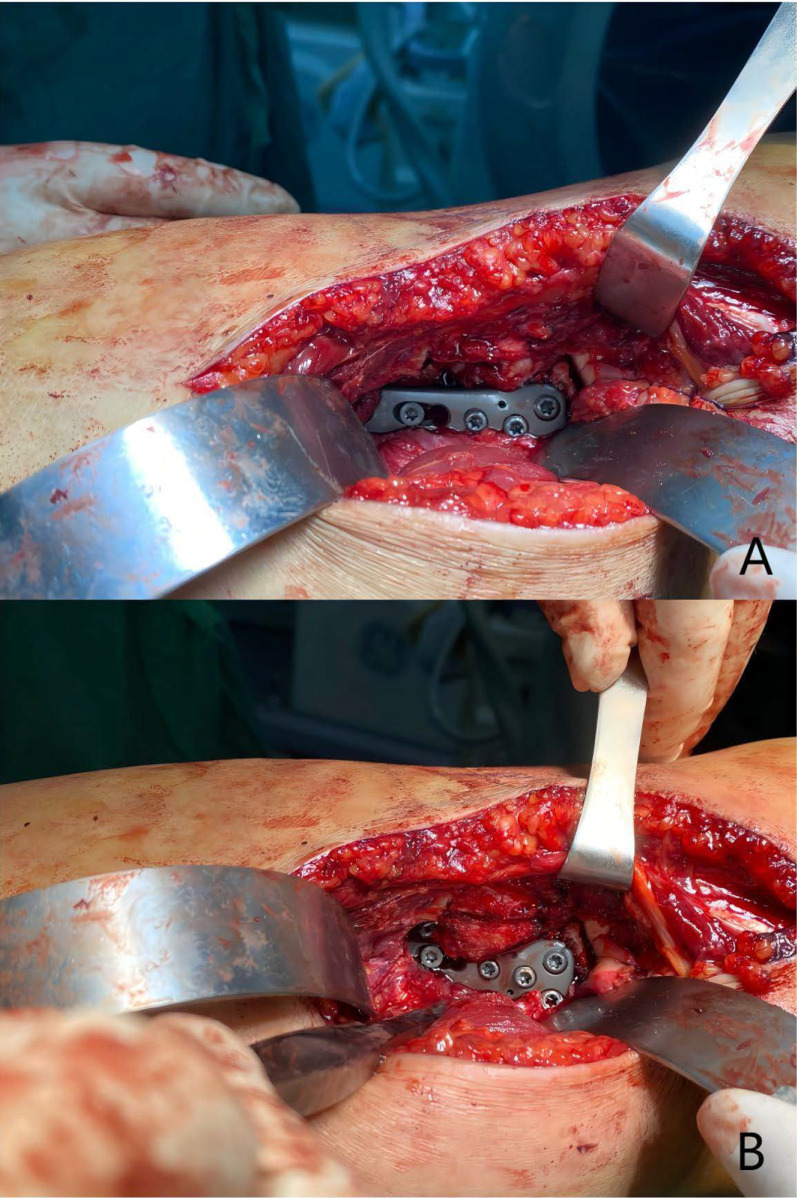
Repositioning of the fibular head osteotomy segment. **A** The plate is placed along the posterior edge of the fibular head to avoid obstructing the repositioning of the osteotomy segment. **B** The fibular osteotomy segment is successfully repositioned

The common peroneal nerve was retracted anteriorly to keep it away from the osteotomy site. An osteotomy line was drawn from the posterior edge of the biceps femoris tendon insertion to the fibular neck, with a perpendicular line at the fibular neck–diaphysis junction. A micro-osteotome was used to perform the osteotomy along this line. The osteotomy area was an exposed region without soft tissue attachment, with an approximate size of 2 × 1.5 cm (Figs. 2, 3). After removing the osteotomized segment, the entire posterolateral column of the tibial plateau was exposed (Fig. 4). Intraoperatively, the posterior horn of the lateral meniscus was often found to be compressed into the fracture site and was retracted to expose the articular surface. Under direct visualization, the fracture fragments were reduced using a periosteal elevator, with temporary fixation achieved using 1.5 mm Kirschner wires. C-arm fluoroscopy was used to confirm articular surface congruence, after which a 3.5 mm-thick buttress plate was applied for definitive fixation. Care was taken to ensure that the proximal portion of the plate did not obstruct the fibular head region, allowing for proper replacement of the osteotomized segment. The fibular head was then restored and fixed with a 3.5 mm cortical screw (Fig. 5). Hemostasis was achieved, the surgical site was irrigated, and the posterior joint capsule and fibular attachment of the soleus were repaired. A vacuum drainage tube was placed within the wound, and the incision was closed in layers and dressed. The procedure was completed.

### Postoperative management

All patients received prophylactic cephalosporin antibiotics within 24 h after surgery to prevent infection. Routine administration of low-molecular-weight heparin calcium was initiated 8 h postoperatively to prevent deep vein thrombosis. After anesthesia recovery, patients were instructed to perform isometric lower limb muscle exercises. On the third postoperative day, patients were encouraged to begin active and passive knee joint exercises, ranging from 0° extension to 45° flexion, with gradual increases in range as tolerated. Follow-up radiographs of the knee in anteroposterior and lateral views were taken at 1, 2, 3, 6, and 12 months postoperatively. Patients were guided to begin partial weight-bearing ambulation with crutches at 1 month postoperatively, progressing to full weight-bearing ambulation at 3 months postoperatively.

## Results

**Fig. 6:**
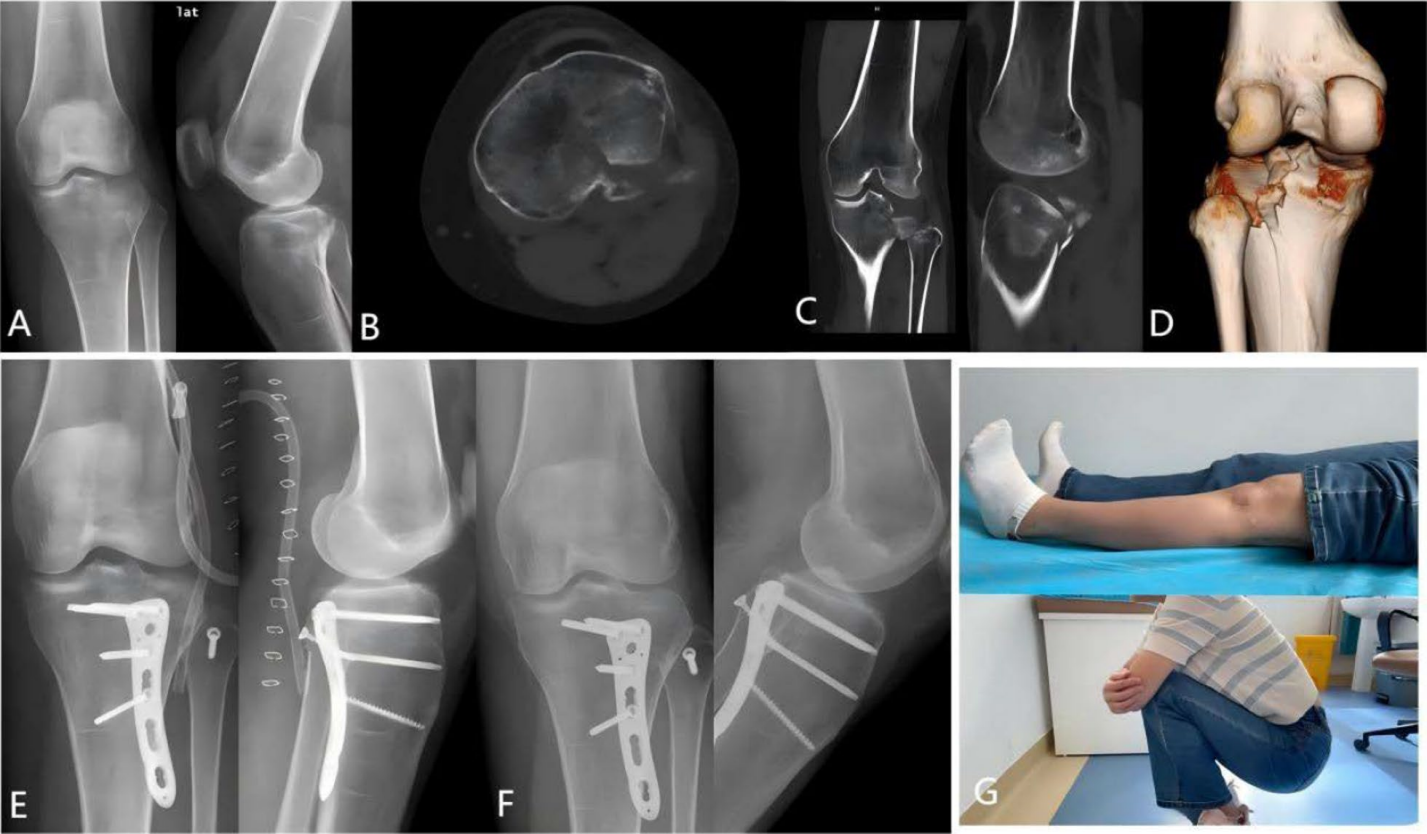
A case of a posterolateral tibial plateau fracture classified as Type III PL according to the Kfuri–Schatzker classification. **A** Preoperative anteroposterior and lateral X-rays of the affected knee joint. **B**–**D** Preoperative CT scans with 3D reconstruction showing significant depression of the posterolateral articular surface and rupture of the posterior wall. **E** Postoperative anteroposterior and lateral X-rays of the knee joint showing perfect fracture reduction. **F** Anteroposterior and lateral X-rays at 6 months postoperatively showing healed fracture and osteotomy sites. **G** Clinical photograph at 1 year postoperatively demonstrating good functional recovery of the knee joint

**Fig. 7:**
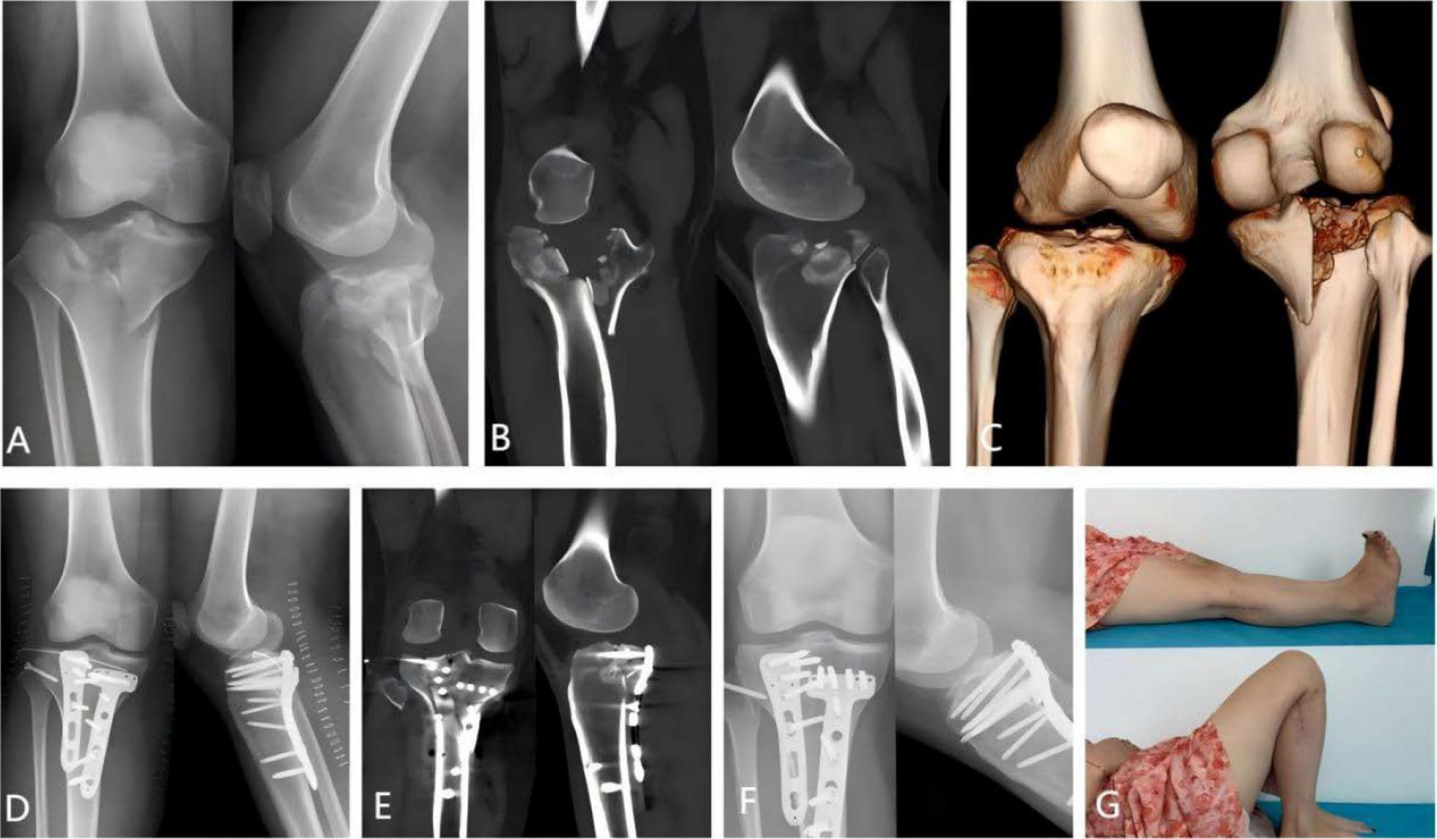
A patient with bilateral tibial plateau fractures, classified as Type V PL + PM + AM according to the Kfuri–Schatzker classification. **A** Preoperative anteroposterior and lateral radiographs of the affected knee. **B**, **C** Preoperative CT scans and 3D reconstruction of the affected knee, showing significant collapse of the posterolateral joint surface, rupture of the posterior wall, and fracture of the medial column. **D** Postoperative anteroposterior and lateral radiographs of the affected knee, demonstrating perfect fracture reduction. **E** Postoperative CT scans and 3D reconstruction of the affected knee, showing excellent fracture reduction. **F** Anteroposterior and lateral radiographs at 6-month follow-up, indicating complete healing of the fracture and osteotomy site. **G** Radiographs of the affected knee at 1-year follow-up, showing excellent recovery of knee function. *AM* anteromedial, *PM* posteromedial

A total of 13 patients were included in this study, with a mean follow-up duration of 12.2 months (range: 9–17 months). On the basis of the Kfuri–Schatzker classification, 30.8% (*n* = 4) of patients were classified as Type III P fractures, characterized by isolated posterolateral tibial plateau compression fractures, while 46.2% (*n* = 6) were bicondylar fractures, involving posterolateral tibial plateau fractures combined with medial column fractures, as shown in Table 2. The degree of posterolateral fracture compression was assessed on sagittal CT scans. The mean preoperative compression was 11.8 ± 4.8 mm (range: 5.2–21.3 mm), while the mean postoperative compression was 0.4 ± 0.8 mm (range: 0–2.5 mm). The mean operative time was 151.9 ± 37.1 min (range: 100–240 min), and the mean intraoperative blood loss was 150.0 ± 84.2 mL (range: 50–300 mL). The mean Rasmussen radiological score was 15.5 ± 2.5 (range: 10–18), with four cases rated as excellent, eight as good, and one as fair. The mean knee flexion range was 142.3° ± 6.0° (range: 125°–145°), and the mean knee extension range was −2.7° ± 3.3° (range: −5° to 5°). The mean Hospital for Special Surgery (HSS) score was 89.8 ± 6.4 (range: 78–98) (Table 3), with 10 cases rated as excellent and 3 as good. All fractures and fibular head osteotomy sites achieved complete healing without any cases of surgical site infection or common peroneal nerve injury (Figs. 6, 7).

**Table 2:** Distribution of the patients according to the Kfuri–Schatzker classification

Kfuri–Schatzker	*n* (%)
Type II, PL	2 (15.3%)
Type III, P	1 (7.7%)
Type III, PL	4 (30.8%)
Type V, PL + PM	2 (15.4%)
Type V, P + PM	2 (15.4%)
Type V, PL + PM + AM	2 (15.4%)

*P* posterior, *PL* posterolateral, *AM* anteromedial, *PM* posteromedial

**Table 3:** Surgical and clinical outcomes

Patients	Operation time (min)	Blood loss (mL)	Preoperative depression (mm)	Postoperative depression (mm)	Rasmussen radiological scores	Follow-up (months)	Extension (°)	Flexion (°)	HSS score
1	160	200	21.3	0	16	9	−5	145	96
2	155	200	15.5	0	18	13	−5	145	94
3	160	150	13.2	0	18	15	−5	145	97
4	170	300	10.2	0	16	10	−5	145	86
5	240	100	17.2	0	12	10	−5	145	87
6	130	100	6.2	0	16	12	0	140	92
7	140	100	15.3	0	18	17	0	145	98
8	135	50	6	0	16	12	0	145	89
9	160	100	10.5	0	18	12	−5	145	94
10	120	200	7.7	1	14	11	0	135	84
11	110	50	11.6	2	14	13	−5	145	80
12	195	300	13.2	2.5	10	11	5	125	78
13	100	100	5.2	0	16	14	−5	145	92
Mean	151.9 ± 37.1	150.0 ± 84.2	11.8 ± 4.8	0.4	15.5 ± 2.5	12.2	−2.7 ± 3.3	142.3 ± 6.0	89.8 ± 6.4

## Discussion

The posterolateral tibial plateau fracture is often caused by knee flexion injuries, typically presenting as compression fractures. When the posterior wall is fractured, it can lead to complex intra-articular comminuted fractures of the joint surface [18]. For displaced posterolateral tibial plateau fractures, achieving anatomical reduction of the joint surface and stable fixation is crucial for maintaining knee range of motion and stability. One of the major factors contributing to poor joint reduction in complex tibial plateau fractures is limited visualization of the tibial plateau joint surface during surgery [19]. This is particularly true for posterolateral tibial plateau fractures, where the fibular head and common peroneal nerve obstruct direct exposure, making reduction and fixation more challenging. As such, surgery in this region remains a significant challenge for orthopedic surgeons.

The choice of surgical approach can directly affect the quality of fracture reduction, the stability of fracture fixation, and postoperative rehabilitation. In recent years, many researchers have explored non-osteotomy approaches for treating posterolateral tibial plateau fractures. For instance, Frosch et al. proposed a new surgical approach that involves freeing the common peroneal nerve and using two windows to expose the anterolateral and posterolateral joint surfaces, allowing for simultaneous reduction and fixation of both the lateral and posterior tibial plateau fractures. However, this approach remains limited due to the obstruction of the fibular head, particularly in visualizing the far posterolateral region, and it can also affect the placement of the support plate [1]. Carlson et al. [20] used a direct posterolateral approach for treating posterolateral tibial plateau fractures, but this approach is unsuitable for complex, comminuted fractures due to the obstruction caused by the fibular head. Yu et al. [21] employed a new lateral approach for the reduction and fixation of posterolateral compression fractures, but this approach does not allow for adequate visualization of the posterolateral region, and thus cannot be used for fractures with cortical disruption of the posterolateral wall.

Common osteotomy approaches include fibular head–neck osteotomy and femoral condyle osteotomy. Durigan et al. [8] used an extended anterolateral approach with femoral condyle osteotomy to treat posterolateral tibial plateau fractures. This approach allowed for direct reduction and strong fixation of the fracture without compromising knee joint function. However, it is not suitable for comminuted fractures of the posterolateral region and may lead to posterolateral rotational instability and intra-articular damage. A recent cadaveric study [22] demonstrated that the visibility of the joint with fibular neck osteotomy was significantly better than with femoral condyle osteotomy, but it increased the potential risk of injury to the common peroneal nerve and the tibiofibular joint, and sometimes does not require such extensive joint exposure. Yu et al. [23] applied a fibular head osteotomy approach to treat posterolateral tibial plateau fractures and achieved good fracture reduction and fixation. Chen et al. [5] suggested that fibular head osteotomy for posterolateral tibial plateau fractures provides excellent exposure of the posterolateral joint surface without any adverse effects. Yao et al. [12] employed a V-shaped fibular head osteotomy to treat posterolateral tibial plateau fractures, concluding that it allowed complete exposure of the fracture and provided stable fixation. Although these studies utilizing fibular head osteotomy approaches have shown good clinical outcomes, there are still significant limitations. First, this approach disrupts the structure of the proximal tibiofibular joint, potentially leading to knee instability [24, 25]. Second, there is an increased risk of common peroneal nerve injury [4, 26]. Additionally, because of the disruption of the biceps femoris tendon, the risk of postoperative knee stiffness is increased [12].

In this study, all 13 cases of posterolateral tibial plateau fractures presented with disruptions of the posterolateral wall boundary and comminution of the posterolateral articular surface, classifying them as complex posterolateral fractures. To address these challenges, we developed a partial fibular head osteotomy approach. This approach requires the dissection and protection of the common peroneal nerve prior to the osteotomy. During surgery, only the posterior portion of the fibular head is removed. Although the osteotomy fragment includes part of the articular surface, it does not completely disrupt the proximal tibiofibular joint, thus preserving its stability. Additionally, the biceps femoris tendon attachment is maintained, reducing the risk of postoperative knee stiffness. Notably, the osteotomy region is distinct from the area of the common peroneal nerve, allowing the nerve to be safely retracted during the procedure without risk of damage. In this study, as all tibial plateau fractures involved only the posterolateral or posteromedial regions, we adopted a modified Frosch approach, which requires exposure through a posterior “window.” Removing the osteotomy fragment provided full exposure of the posterolateral region of the tibial plateau, enabling unobstructed visualization for the reduction and fixation of complex comminuted fractures, particularly those involving the extreme posterolateral region and the disrupted posterolateral wall. Following fracture reduction, the support plate was strategically placed along the posterolateral margin of the fibular head to avoid obstructing the repositioning of the osteotomy fragment and to facilitate future plate removal. For extreme posterolateral fracture fragments, additional fixation using 1.5 mm Kirschner wires was required to ensure adequate stability. In our cohort, no cases of knee instability or common peroneal nerve injury were observed. The quality of fracture reduction, as assessed by the Rasmussen score (15.5 ± 2.5), and postoperative knee function, as measured by the HSS score (89.8 ± 6.4), were comparable to or superior to those achieved with the complete fibular neck osteotomy approach [4, 9]. Additionally, the improvement in knee range of motion (flexion: 142.3° ± 6.0°; extension: −2.7° ± 3.3°) was superior to that reported for other partial fibular head osteotomy approaches [12, 23, 27].

Although this study had a relatively small sample size of only 13 patients, it included an adequate average follow-up period and comprehensive postoperative evaluations, including both clinical and radiological assessments. The findings demonstrate the feasibility of this technique, particularly for complex comminuted fractures of the posterolateral tibial plateau. This approach enables optimal fracture reduction and fixation without compromising knee joint function. However, we acknowledge that due to the small sample size and relatively short follow-up period in this study, this technique may still present unpredictable risks for patients. Additionally, performing this surgical approach requires a deep understanding of the posterolateral knee anatomy. Precise intraoperative dissection of the common peroneal nerve is necessary, and due to the proximity of the popliteal vascular structures, the procedure demands a high level of surgical expertise and extensive experience.

## Limitations

The limitations of our study include its retrospective design, small sample size, and short follow-up duration. Therefore, future large-scale, prospective, randomized controlled studies with extended follow-up periods are needed to further validate our findings.

## Conclusions

In summary, the partial fibular head osteotomy approach, preserving the biceps femoris tendon attachment, provides excellent visualization of the posterolateral tibial plateau, facilitating direct fracture reduction and fixation. This technique carries a low risk of common peroneal nerve injury and does not compromise knee joint stability. Therefore, we recommend this surgical method for the treatment of complex posterolateral tibial plateau fractures.

## Data Availability

All data generated or analyzed during this study are included in this published article.

## Abbreviations

HSS: Hospital for Special Surgery
P: Posterior
PL: Posterolateral
AM: Anteromedial
PM: Posteromedial
